# Incidence of Subclinical Deep Vein Thrombosis after Total Hip and Knee Arthroplasty Is Not Correlated with Number of Tranexamic Acid Doses

**DOI:** 10.3390/jcm13133834

**Published:** 2024-06-29

**Authors:** Bogdan-Axente Bocea, Bianca-Iulia Catrina, Mihai-Dan Roman, Nicolas Catalin Ionut Ion, Sorin Radu Fleaca, Cosmin-Ioan Mohor, Antonescu Oana Raluca, Sergiu-Ioan Moga, Romeo Gabriel Mihaila

**Affiliations:** 1 Faculty of Medicine Sibiu, Lucian Blaga University, Str. Lucian Blaga Nr. 2A, 550169 Sibiu, Romania; bogdanaxente.bocea@ulbsibiu.ro (B.-A.B.); nicolascatalinionut.ion@ulbsibiu.ro (N.C.I.I.); radu.fleaca@ulbsibiu.ro (S.R.F.); cosmin.mohor@ulbsibiu.ro (C.-I.M.); oanaraluca.antonescu@ulbsibiu.ro (A.O.R.); romea.mihaila@ulbsibiu.ro (R.G.M.); 2 County Clinical Emergency Hospital, 550245 Sibiu, Romania; mogasergiu1720@yahoo.com

**Keywords:** tranexamic acid, TXA, varying doses, THA, TKA

## Abstract

**Background:** Recent studies increasingly highlight the efficacy of tranexamic acid administration in total hip arthroplasty (THA) and total knee arthroplasty (TKA). However, the optimal dosage of tranexamic acid is still controversial. **Methods:** The current study analyzes the efficiency of tranexamic acid dosage and the number of administrations in THA and TKA. The objective of this study is to compare the incidence of deep vein thrombosis (DVT) based on the number of dosages. We divided the patients into two groups; one group received a single dosage, and the other group received two dosages. Doppler ultrasound examinations were conducted on the lower limbs of all patients at both six and thirty days postoperatively. The second objective is to compare the decrease in hemoglobin (Hb) in the two groups. **Results:** The results show that there is no difference in DVT incidence between the patients with different TXA numbers of dosages. There is no statistically significant decrease in Hb between the two groups at day one and day five postoperatively. Day one shows a statistically higher average in the two-dose group, approximately 0.06 g/dL, and day five shows a slightly elevated average in the single-dose group, approximately 0.06 g/dL. Blood transfusion requirements show no significant differences in the groups; one patient in the single-dose tranexamic acid group needed transfusion at day five postoperatively, while two patients in each group required immediate postoperative transfusion. **Conclusion:** There was no increase in the incidence of deep vein thrombosis among patients receiving two dosages of tranexamic acid.

## 1. Introduction

There was a notable increase in osteoarthritis in the United States between 1996 and 2006, from approximately 14 million individuals to reaching a total of 90 million in 2006 from the 76 million reported in 1996. This is a trend commonly attributed to the aging demographic of the population [1,2].

The occurrence of deep vein thrombosis continues to be a significant postoperative concern following hip and knee replacement surgeries, primarily due to the associated risk of pulmonary embolism (PE). The reported incidence of PE varies, ranging from 0.9% to 28% for hip arthroplasty and 1.5% to 10% for knee arthroplasty. Both immobilization and surgical interventions contribute to an elevated risk of clot formation, with a concomitant 1.9% risk of heart attack following hip arthroplasty [3,4]. The risk of pulmonary embolism persists, particularly in the context of TKA, where the risk of DVT within 90 days post-surgery can reach 4% [5]. The diagnosis and management of thrombotic events after these surgical procedures present ongoing challenges, and it is noteworthy that no singular treatment modality completely eliminates the associated risk [6].

Recent research has highlighted the efficacy of tranexamic acid (TXA) during and after THA and TKA. Patients administered TXA exhibited a reduced requirement for blood transfusions and experienced fewer post-surgery complications [7].

In a study conducted by Feng-Chih Kuo and colleagues, published in September 2018 and involving 930 patients, those who received TXA demonstrated a significantly diminished need for transfusions [8].

Another study reported a lower infection rate in patients treated with TXA. Intriguingly, even individuals with coagulation issues or a history of heart attacks did not face substantial risks associated with TXA administration [9,10].

Regarding the optimal dosage of tranexamic acid (TXA), numerous studies have sought to determine the most efficacious quantity. A 2020 investigation indicated that multiple doses were more effective in preserving hemoglobin levels, yet it did not compare blood transfusion rates between single and multiple doses. While other studies have established the high effectiveness of TXA, there remains a gap in evidence regarding the superiority of multiple doses over single doses and the relative merits of oral versus intravenous administration [11,12,13].

In 2014, a comparative study evaluating two anticoagulants revealed a higher incidence of deep vein thrombosis in knee arthroplasty patients compared to those undergoing hip arthroplasty, as assessed by Doppler ultrasound [14].

This study was designed to investigate the dose-dependent effectiveness of tranexamic acid in patients undergoing hip and knee replacement surgery and to study whether patients who receive two doses have a higher risk of developing DVT. A bilateral Doppler ultrasound of the lower limbs was performed for all patients for the identification of subclinical DVT.

The Hb level was determined at days one and five postoperatively in order to evaluate if the number of TXA doses influences blood loss. At the same time, we followed the transfusion rate in the two groups.

## 2. Materials and Methods

This prospective randomized study initially enrolled forty patients treated consecutively between June and July 2023 in the Orthopedics and Traumatology Department at the Sibiu County Emergency Hospital, all of whom underwent either total hip arthroplasty or total knee arthroplasty [15]. This study was approved by the Ethics Committee of the hospital.

A single preoperative dose, administered as a 500 mg/5 mL ampoule thirty minutes before surgery, is comparable in efficacy to the administration of two doses one preoperatively (as in a single dose) and one postoperatively—eight hours after surgery, each consisting of a 500 mg/5 mL dose. TXA was administrated only intravenously. The first admitted patient received two doses, the second patient received one dose, and so on, alternating between one dose and two doses, resulting in twenty patients who received a single preoperative dose of TXA and twenty patients who received two doses of TXA. 

Exclusion criteria were patients with a history of DVT and TVP, patients who presented an infection [16], patients with anticoagulant treatment for cardiology pathology, patients with preoperative hemoglobin lower than 11 g/dL for women and lower than 13 g/dL for men, and patients inadequately screened during the postoperative (PO) period. During the follow-up, two patients from the two-dose group were lost; they did not show up at the 30-day PO follow-up.

We conducted a postoperative (PO) follow-up on patients using a bilateral Doppler ultrasound of the lower limbs to facilitate the early detection of deep vein thrombosis (DVT). This investigation aimed to discern whether variations in the incidence of DVT could be observed based on the administered tranexamic acid dosage. All examinations were conducted utilizing the Philips Diagnostic Ultrasound System, Model Epiq 7, under consistent conditions maintained by the same physician. Follow-up assessments occurred on PO days 6 and 30, with our primary endpoints focusing on venous thrombosis (VT) and venous thromboembolic events (VTEs).

Diagnostic criteria for VT included characteristics such as the inability to close the venous lumen, hypoechoic or echoless cavity, minimal or absent blood flow signal within the thrombosis, and a Doppler pulse indicating either no blood flow or a lack of change with respiration. In all patients within this group, antithrombotic prophylaxis consisted of Clexane, administered as a 6000 IU (60 mg/0.6 mL) subcutaneous injection. Initiation commenced one day before surgery, continuing for twenty-five days postoperatively.

Hemoglobin samples were collected on the day before the surgery and on days one and five after surgery. During surgery, two doses of Lidocaine 10 mg/mL and one dose of Adrenaline (Epinephrine) 1 mg/mL were administered intra-articularly, after surgery, no drainage was used, and during TKA surgery, a tourniquet was used. Patients were mobilized immediately postoperatively, with a weight-bearing exercise (using a walker) and a passive range of motions in consultancy with the Department of Rehabilitation. Wound dressing was performed on days 1, 3, and 14 after surgery. The wound was monitored to evaluate superficial infection, hematoma or seroma, major bruises, oozing, and blisters.

For the statistical analysis of the obtained results, we used the SPSS Statistic 28.0 software. The quantitative parameters were described as the means ± standard deviations, and the qualitative parameters were expressed as frequencies or percentages. All tests were two-sided, and *p* values less than 0.05 were considered statistically significant.

## 3. Results

The patients enrolled in the study ranged in age from 52 to 87; there were 22 females and 16 men, with an average BMI of 33.165. The distribution by groups is provided in Table 1.

The use of two doses of intravenous tranexamic acid did not increase the likelihood of experiencing total venous thromboembolic events (VTEs). A 66-year-old female was found to have partial femoral vein thrombosis by Doppler ultrasonography exams, at PO day 6. Treatment with Rivaroxaban was administered on the first 7 days, with two doses of 15 mg per day followed by a treatment with a single daily dose of 20 mg. At the 30-day PO follow-up, there were no signs of venous thrombosis. Only one dose of TXA was administrated to this female. This condition was observed in just 1.28% of cases.

The statistical analysis of the hemoglobin readings after surgery showed no significant differences between the groups. The average reduction in hemoglobin levels 24 h after surgery was 1.89 g/dL in the single-dose tranexamic acid group and 1.85 g/dL in the two-dose group. The extent of hemoglobin loss showed similar extremes in both groups. In the single-dose group, the highest decrease was 3.7 g/dL, but in the two-dose group, it was 3.9 g/dL. In contrast, the smallest reduction in the group receiving a single dose was 0 g/dL, suggesting that some patients maintained a stable hemoglobin level twenty-four hours after the surgery (specifically at 13.9 g/dL). In the group that received two doses, the least decrease was 0.7 g/dL, observed twenty-four hours after the surgery (Table 2 and Table 3).

An analysis of the decrease in hemoglobin levels five days after surgery, compared to the baseline values after twenty-four hours, showed similar patterns. In the single-dose tranexamic acid group, the average decrease in hemoglobin was 0.87 g/dL, while in the two-dose group, it was 0.855 g/dL. The results from the five-day assessment showed that the group receiving a single dosage of tranexamic acid experienced a significant decrease in hemoglobin levels, with a loss of 2.8 g/dL being the most prominent. Remarkably, there was one exceptional case that diverged from this pattern, exhibiting a 0.5 g/dL rise in hemoglobin levels five days after the surgery, as opposed to twenty-four hours postoperatively. The group that received a double dosage of tranexamic acid experienced a maximum reduction of 2.5 g/dL after five days. Similar to the group that received a single dose, one patient exhibited a 0.5 g/dL rise in hemoglobin levels at the five-day point, in comparison to the levels observed twenty-four hours after the surgery (Table 2 and Table 3).

Within both groups, a subset of patients required blood transfusions. In the single-dose tranexamic acid group, three patients, all male, received transfusions, two of whom were transfused immediately after surgery by interventional therapy staff, while one patient required a transfusion after the five-day control hemoglobin measurement. Similarly, in the double-dose tranexamic acid group, two patients, both female, necessitated transfusions, both administered promptly after surgery by the ICU staff. Although there were differences in the extent of hemoglobin decline and occurrences of both decreases and increases after five days, both groups showed a clear need for no blood transfusions, albeit with slightly varied administration patterns (Table 4).

There were no major wound complications, and no patients needed extra dressing or wound care for superficial infections, hematomas, or seromas. No significant intergroup difference was observed.

## 4. Discussion

The results of our study show that TXA use does not significantly increase the incidence of symptomatic venous thrombosis. TXA utilization in total knee arthroplasty, as a routine method for preventing anticoagulation, does not lead to a heightened incidence of postoperative symptomatic VTE. In our group, the incidence of DVT is only 1.28% compared to other studies where it is higher, around 8.6% [17]. In another study comparing the incidence of DVT in patients given intra-articular TXA and those not given TXA, the rate of DVT was 4.3%. This study shows, like our study, that there is no difference in the incidence of DVT between the two groups [18].

The patient diagnosed with deep vein thrombosis (DVT) received treatment with Rivaroxaban, initially at 15 mg twice a day for 7 days and subsequently at 20 mg once a day for 23 days. At the 30-day postoperative assessment, there were no signs of venous thrombosis (VT) or venous thromboembolic events (VTEs). Notably, this patient, who had the highest BMI among all participants, was in the group that received only one dose of tranexamic acid (TXA), while all patients receiving two doses remained free from VT.

BMI is recognized as an independent predictor for VT; it is inconclusive whether TXA administration plays a decisive role in thrombotic events [19]. Additionally, numerous studies have investigated VT incidence following orthopedic procedures. In one study by Bin Abd Razak et al. [20], among 531 Asian patients undergoing unilateral total knee arthroplasty without postoperative anticoagulation, only 4 individuals (0.75%) experienced symptomatic VTE, including one case of symptomatic pulmonary embolism. Conversely, a meta-analysis by Januel et al. [21] involving 44,844 instances of total hip arthroplasty or total knee arthroplasty, incorporating anticoagulation, reported overall occurrence rates of symptomatic VTE, deep vein thrombosis, and pulmonary embolism after total knee arthroplasty at 1.09%, 0.63%, and 0.27%, respectively.

In a retrospective study by Poeran et al. [22] covering 870,000 patients undergoing hip or knee replacement over six years in 510 U.S. hospitals, deep venous thrombosis occurred in 0.4% of TXA-receiving patients and slightly higher at 0.5% in those not receiving TXA. Furthermore, a substantial body of the literature indicates that TXA has a satisfactory safety profile, with no observed increase in the risk of VTE [23,24,25,26,27,28]. These findings are similar to the result that we obtained.

The comparative analysis of hemoglobin levels at day one postoperatively revealed a relatively uniform decline in both study groups. A marginal difference of approximately 0.04 g/dL was observed, indicating a slightly higher average decrease in hemoglobin among patients in the two-dose tranexamic acid group compared to the single-dose group. The average decline after day one PO was 1.89 g/dL for patients receiving one dose and 1.85 g/dL for patients with two doses. These changes in the Hb level are comparable with other studies found in the literature [29].

This consistency in hemoglobin reduction persisted at the day five postoperative assessment, with the single-dose tranexamic acid patients displaying a slightly higher average decrease of approximately 0.015 g/dL. The average decline after PO day five was 0.87 g/dL for patients receiving one dose and 0.855 g/dL for patients receiving two doses. The double-dose group exhibited a comparable decline, reinforcing the consistent response to the drug across both groups. These results are different than the findings in other studies that followed the Hb level for five days [11].

Our study found that there is no difference observed between the two groups in Hb decline at PO day one and also at PO day five, as some studies in the literature show. The study by Andrew G Golz conducted on a group of approximately 1500 patients divided into two groups as in our study showed that there were no differences between the group that received one dose and the group that received two doses [30]. This is different from other studies in the literature where there was a difference. The study conducted by Xiang-Dong Wu on a group of approximately 360 patients shows that with multiple doses, the Hb level decrease is lower [11].

Another finding of this study is that there is no difference in blood transfusion requirements. Both groups demonstrated a similar pattern, with two patients each requiring immediate postoperative transfusions. The only difference was from a demographic point of view; in the group with two doses, there were two women who needed blood transfusion, while in the group with one dose, two men needed blood transfusion. At the five-day postoperative interval, only one patient necessitated a transfusion, in the single-dose tranexamic acid group. This patient also had the greatest decrease in the Hb value between days one and five with 2.8 points. Blood transfusion requirements were very low, with only 13% of the patients needing blood transfusion, confirming data from the literature [10]. Our study shows that there is no significant difference between these two groups, compared to other studies that compared one dose with two doses, one dose administrated intravenously and one intra-articularly [31].

We found no significant difference between the two groups in terms of wound complications such as superficial infections, hematomas, or seromas. These findings are comparable with data from other studies that conducted a follow-up of the PO wound in patients receiving TXA [32].

The strengths of this study include that it is a prospective study on a randomly selected group of patients. Subclinical DVT was also investigated, not only in symptomatic patients. The follow-up was until PO day 30, a few days after the anticoagulant protection ended. 

A limitation of this study is that we had a reduced number of patients. 

## 5. Conclusions

This study shows that there is no increased incidence of DVT among patients who received two doses of TXA compared with a single dose.

The findings of this study suggest that the administration of a second dose of tranexamic acid (TXA) may not be necessary, as shown by the values of both hemoglobin levels and blood transfusion requirements.

## Figures and Tables

**Table 1:** Demographic distribution.

Dosage	THA/TKA	Age	Sex (F/M)	BMI
entry 1	12/8	67.5	13/7	33.175
entry 2	10/8	72	8/10	33.155

**Table 2:** Differences in Hb value according to dose.

	Dosage	N	Average	Std. Deviation
difference between Hb before and day 1	1 dose	20	1.8900	1.02130
	2 doses	18	1.8500	0.85492
difference between Hb day 1 and day 5	1 dose	20	0.8700	0.72264
	2 doses	18	0.8556	0.72940

**Table 3:** Statistical difference in Hb according to dose.

		*t*-Test for Equality of Means						
		pp	df	ig. (2-Tailed)	Mean Dif.	Std. Error Dif.	95% Confidence Interval of the Dif.	
							Lower	Upper
Hb day 1	Equal variances assumed	−0.377	36	0.708	−0.16389	0.43480	−1.04570	0.71792
	Equal variances not assumed	−0.375	34.776	0.710	−0.16389	0.43667	−1.05059	0.72281
Hb day 5	Equal variances assumed	−0.376	36	0.709	−0.17333	0.46102	−1.10833	0.76166
	Equal variances not assumed	−0.373	34.032	0.711	−0.17333	0.46436	−1.11699	0.77032

**Table 4:** Blood transfusion requirement according to dose.

		Frequency	Percent	Valid Percent
**Valid**	yes	5	13.2	13.2
	no	33	86.8	86.8
	Total	38	100.0	100.0

## Data Availability

The data that support the findings of this study are available from the corresponding author upon reasonable request.
